# Handgrip weakness, low fat‐free mass, and overall survival in non‐small cell lung cancer treated with curative‐intent radiotherapy

**DOI:** 10.1002/jcsm.12526

**Published:** 2020-02-11

**Authors:** Chris Burtin, Jacques Bezuidenhout, Karin J.C. Sanders, Anne‐Marie C. Dingemans, Annemie M.W.J. Schols, Stephanie T.H. Peeters, Martijn A. Spruit, Dirk K.M. De Ruysscher

**Affiliations:** ^1^ REVAL—Rehabilitation Research Center, BIOMED—Biomedical Research Institute, Faculty of Rehabilitation Sciences Hasselt University Diepenbeek Belgium; ^2^ Department of Radiation Oncology (MAASTRO Clinic) Maastricht University Medical Centre, GROW School for Oncology and Developmental Biology Maastricht The Netherlands; ^3^ Department of Respiratory Medicine Maastricht University Medical Centre, NUTRIM School of Nutrition and Translational Research in Metabolism Maastricht The Netherlands; ^4^ Department of Respiratory Medicine Maastricht University Medical Centre, GROW School for Oncology and Developmental Biology Maastricht The Netherlands; ^5^ Department of Research & Development CIRO Horn, The Netherlands

**Keywords:** Non‐small cell lung cancer, Handgrip strength, Fat‐free mass, Muscle, Prognosis

## Abstract

**Background:**

Assessment of handgrip strength and fat‐free mass provides quick and objective information on muscle performance and mass that might complement subjective World Health Organization Performance Status (WHO PS). We investigated to what extent the presence of pre‐treatment handgrip weakness and low fat‐free mass index (FFMI) provides additional prognostic information on top of well‐established prognostic factors (including WHO PS) in non‐small cell lung cancer (NSCLC) patients selected for curative‐intent (chemo)radiation.

**Methods:**

Prospectively, patients with early and locally advanced NSCLC (stages I‐III) treated with (chemo)radiation were enrolled. Handgrip weakness and low FFMI, derived from bioelectrical impedance analysis, were defined using normative values and were correlated with overall survival (OS).

**Results:**

We included 936 patients (age 68 ± 10 years; 64% male; 19% stage I, 9% stage II, and 72% stage III disease; 26% handgrip weakness; 27% low FFMI). In patients with good performance status (WHO PS 0 or 1), handgrip weakness and low FFMI were significant prognostic factors for OS, after adjustment for age, gender, disease stage, and co‐morbidities. The combined presence of handgrip weakness and low FFMI was a strong prognostic factor for OS when compared with patients with normal handgrip strength and FFMI (hazard ratio: 1.79, 95% confidence interval: 1.34–2.40, *P* < 0.0001). In patients with impaired performance status (WHO PS ≥ 2, 19% of sample), handgrip weakness and low FFMI were not related to OS.

**Conclusions:**

In early and locally advanced NSCLC patients treated with curative‐intent (chemo)radiation who have good WHO PS, patients with combined handgrip weakness and low FFMI have the worst prognosis.

## Introduction

Lung cancer is amongst the biggest killers worldwide, accounting for 1.7 million deaths annually.^1^ Although survival rates are improving because of advanced diagnostic strategies and new effective treatment modalities, mortality rates continue to be high.

Adequate scoring of patients' performance status is important for assessment of prognosis and to determine the optimal therapeutic approach. In routine clinical practice, performance status is commonly estimated using the World Health Organization Performance Status (WHO PS), which is a doctor‐reported outcome.^2^ It is likely that doctors underestimate or overestimate the patient's true performance status.^3^ Also, in patients with a good physical performance status (WHO PS 0–1), outcome is still heterogeneous.^4^ Therefore, a more objective evaluation of patient's performance status may complement or substitute WHO PS.

Computed tomography‐derived low muscle mass, as a marker of impaired performance, is a strong prognostic factor for mortality in patients with non‐small cell lung cancer (NSCLC).^5^, ^6^, ^7^ However, ideally, the evaluation of performance status should be cheap, fast, and easy to implement in routine practice. Handgrip strength is a simple and quick measure of muscle function. The maximal handgrip strength is decreased in locally advanced and metastatic NSCLC patients,^8^ and cancer patients with low handgrip strength have worse survival rates.^9^ Next to survival, low handgrip strength is associated with low muscle mass.^9^, ^10^ A simple screening measure for muscle loss is assessing fat‐free mass (FFM) by bioelectrical impedance. To date, information on the prognostic value of maximal handgrip strength and FFM in addition to WHO PS in patients with NSCLC treated with curative intent is lacking.

Our hypothesis was that assessment of handgrip strength and FFM may provide additional prognostic information on top of well‐established prognostic factors (including age, gender, disease stage, and WHO PS) in a large prospective cohort of patients with stage I‐III NSCLC selected for radiotherapy with curative intent.

## Material and methods

### Study design and patients

Between July 2006 and June 2015, a prospective cohort study was conducted at MAASTRO Clinic, Maastricht University Medical Centre, Maastricht, the Netherlands. All consecutive patients with primary NSCLC receiving radiation treatment with curative intent were enrolled. This project was approved by the internal review board of MAASTRO Clinic.

The following parameters were registered: age, gender, TNM classification (based on the TNM classification of malignant tumours 6th edition from 2006 to December 2009, 7th edition thereafter), treatment modality [primary radiotherapy, concurrent chemoradiotherapy (CRT), and sequential CRT; see below for detailed information], Charlson co‐morbidity index, WHO PS, maximal handgrip strength, FFM, weight, height, date of first irradiation, and date of last consultation or date of death. All physical assessments were performed at first consultation for radiation treatment.

Survival was defined as time from date of start of radiation therapy up to death of any cause or date of last visit. Last survival update was August 2018. On this date, survival status of all patients was checked using the electronic records of the Dutch national death registry.

### Measurement protocols

Maximal handgrip strength was measured with a Jamar hydraulic hand dynamometer (JA Preston Corporation, Jackson, MI, USA). The handle was adjusted individually to the size of the patient's hand. The measurement was carried out with the patient seated upright with the arms leaning on the arm‐rests with the elbows in 90° flexion. The patient was instructed to grip the handle with maximal strength during 3 s. The measurements were repeated three times for the left and right hand. This is based on literature suggesting that performing three attempts lowers the risk of misclassification bias (weak vs. not weak) in middle‐aged and older adults.^11^ The highest value for both sides was registered. Patients with handgrip weakness (i.e. maximal handgrip strength below the 10th percentile of the UK Biobank reference values, taking gender, age, and height into account^12^ in at least one side) were identified.

World Health Organization Performance Status was assessed by the radiation oncologist. Patients were grouped based on WHO PS score, that is, patients with a score zero or one vs. patients with score two or higher.^13^ Patients with a score of two or higher were classified to have a low performance status.

The Charlson co‐morbidity index was calculated based on review of individual medical records.^14^

Body height was determined to the nearest 0.5 cm with subjects standing barefoot. Body weight was assessed while subjects wore light clothing and no shoes. Body composition was estimated using single‐frequency (50 kHz) hand‐to‐hand bioelectrical impedance analysis (BIA) (Omron Healthcare Group, Hoofddorp, The Netherlands). Patients were standing with legs apart and arms straight forward, holding the device with both hands. FFM was calculated by subtracting fat mass from total body weight. FFM index (FFMI, kg/m^2^) was calculated by dividing FFM by height squared. Low FFM was defined as an FFMI below 17 kg/m^2^ in male patients and below 15 kg/m^2^ in female patients, in line with the Global Leadership Initiative on Malnutrition criteria for the diagnosis of malnutrition.^15^

### Radiotherapy treatment

All patients underwent a planned 2‐deoxy‐2[fluorine‐18]fluoro‐d‐glucose positron emission tomography integrated with computed tomography scan for delineation of target volumes. Dose prescription was based on the International Commission on Radiation Units 50 and 63 guidelines. Stage I tumours were treated with stereotactic radiotherapy (8 × 7.5 Gy) or fractionated radiotherapy if the tumours were centrally located or overlapped with mediastinal structures (maximally 24 × 2.75 Gy). Stage II tumours were treated with fractionated radiotherapy (24 × 2.75 Gy). Stage III tumours were treated with an individualized isotoxic scheme based on normal tissue constraints. The full and detailed methodology has been published previously.^16^, ^17^

### Treatment

Depending on disease stage and based on patients' overall health status, patients were treated with primary radiotherapy, sequential CRT, or concurrent CRT. Primary radiotherapy was defined as radiotherapy, fractionated radiotherapy, or stereotactic body radiotherapy alone. Patients who received post‐operative radiotherapy after lobectomy or pneumonectomy because of a positive resection margin (R1) and/or persistent N2 disease were excluded from the current analyses. Sequential CRT was defined as chemotherapy followed by radiotherapy with no overlap between the two modalities. Concurrent CRT was defined as treatment with chemotherapy and radiotherapy with any overlap between the two modalities. Generally, patients received one or more cycles of induction chemotherapy (cisplatin/carboplatin and gemcitabine, or cisplatin/carboplatin and etoposide) followed by concurrent CRT. For concurrent chemotherapy and radiotherapy, from 2006 to 2011, chemotherapy consisted of cisplatin and vinorelbine and from 2012 onwards of cisplatin and etoposide.

### Statistical analyses

All statistical analyses were performed with SAS 9.4. Variables were expressed as mean ± standard deviation, median (quartile 1; quartile 3), or in absolute number and proportion, as appropriate. Between‐groups comparisons were performed using independent Student's *t*‐tests for continuous variables or χ^2^ tests for categorical data. Overall survival (OS) was analysed using two‐sided log‐rank, and survival curves were estimated using the Kaplan–Meier method. Non‐proportionality was assessed by log minus log plots and Schoenfeld residuals test. Cox proportional hazard models were used to assess the relationship of handgrip weakness and low FFM with OS during follow‐up in patients with preserved (WHO PS 0 or 1) and impaired (WHO PS ≥ 2) performance status. Hazard ratios (HRs) and their 95% confidence intervals (CIs) were calculated. Analyses were adjusted for age, gender, disease stage, and Charlson co‐morbidity index. Two‐sided *P*‐values lower than 0.05 were considered as statistically significant.

## Results

In total, 1226 NSCLC patients were enrolled in the study. Two hundred ninety patients were excluded because of missing data in one or more of the outcomes used in our model (handgrip strength, *n* = 48; WHO PS, *n* = 87; FFMI, *n* = 90; Charlson co‐morbidity index, *n* = 254). Consequently, 936 patients were eligible for analyses (stage I: *n* = 174; stage II: *n* = 83; stage III: *n* = 679). *Table* 1 summarizes the baseline patient and treatment characteristics. Mean age was 68 years (range 32–91), and 64% were male. Patients received primary radiotherapy (34.4%), concurrent CRT (54.2%), or sequential CRT (11.4%).

**Table 1:** Baseline characteristics

	All patients (*N* = 936)	Stage I (*N* = 174)	Stage II (*N* = 83)	Stage III (*N* = 679)
Age in years, mean (range)	68 (32–91)	72 (42–91)	73 (48–89)	66 (32–89)
>75 years, *N* (%)	226 (24)	71 (41)	37 (45)	118 (17)
Male gender, *N* (%)	598 (64)	101 (57)	60 (72)	437 (65)
WHO PS				
0	221 (24)	31 (18)	15 (18)	175 (26)
1	573 (61)	100 (57)	47 (57)	426 (63)
2	128 (14)	38 (22)	17 (20)	73 (11)
3	13 (1)	5 (3)	4 (5)	4 (1)
4	1 (0)	0 (0)	0 (0)	1 (0)
Histology, *N* (%)				
Adenocarcinoma	215 (23)	20 (12)	16 (19)	179 (26)
Squamous cell carcinoma	334 (36)	39 (22)	38 (46)	257 (38)
Large cell carcinoma	125 (13)	11 (6)	7 (8)	107 (16)
Undifferentiated NSCLC	160 (17)	19 (11)	15 (18)	126 (19)
No histological diagnosis	102 (11)	85 (49)	7 (8)	10 (1)
Charlson co‐morbidity index, mean ± SD	3.2 ± 1.6	4.0 ± 1.3	4.2 ± 1.5	2.9 ± 1.6
Treatment strategy				
Primary radiotherapy	322 (35)	174 (100)	69 (83)	79 (12)
Concurrent CRT	507 (54)	0 (0)	12 (15)	495 (73)
Sequential CRT	107 (11)	0 (0)	2 (2)	105 (15)
BMI in kg/m^2^, mean ± SD	24.9 ± 4.5	25.0 ± 4.8	24.8 ± 4.1	24.9 ± 4.4
FFMI in kg/m^2^, mean ± SD				
Male	18.4 ± 2.2	18.3 ± 2.1	18.0 ± 2.5	18.5 ± 2.2
Female	16.0 ± 2.6	15.7 ± 2.7	15.6 ± 2.8	16.1 ± 2.5
Low FFMI,^a^ *N* (%)				
Male	148 (25)	24 (24)	20 (33)	104 (24)
Female	106 (31)	32 (42)	9 (39)	65 (27)
HGS in kg, mean ± SD				
Right	30.6 ± 10.9	28.6 ± 10.8	27.7 ± 9.9	31.5 ± 10.9
Left	29.4 ± 11.0	27.1 ± 10.8	27.2 ± 10.9	30.2 ± 10.9
Handgrip weakness,^b^ *N* (%)	243 (26)	52 (30)	33 (40)	158 (23)

WHO PS, World Health Organization Performance Status; NSCLC, non‐small cell lung cancer; SD, standard deviation; CRT, chemoradiation; BMI, body mass index; FFMI, fat‐free mass index; HGS, handgrip strength.

a FFMI < 17 kg/m^2^ in male patients and <15 kg/m^2^ in female patients.

b <10th percentile of established normative values [12].

Twenty‐six per cent of the patients showed handgrip weakness at baseline. These weakened patients had worse WHO PS (PS 2 or higher: 28% vs. 11%; *P* < 0.001), were older (71 ± 10 vs. 66 ± 10 years; *P* < 0.0001), were more likely to be male (74% vs. 60%; *P* < 0.001), had a lower body mass index (24.3 ± 4.6 vs. 25.1 ± 4.4 kg/m^2^; *P* = 0.015), a higher likelihood of low FFMI (38% vs. 23%; *P* < 0.0001), and had a higher Charlson co‐morbidity index score (3.8 ± 1.7 vs. 3.1 ± 1.5 points; *P* < 0.0001) compared with patients with a normal handgrip strength.

Twenty‐seven per cent of the patients showed low FFMI at baseline. These patients had worse WHO PS (PS 2 or higher: 22% vs. 12%; *P* < 0.0002), were more likely to be female (42% vs. 34%; *P* < 0.029), had a lower body mass index (20.9 ± 2.8 vs. 26.4 ± 4.0 kg/m^2^; *P* = 0.015), a higher likelihood of handgrip weakness (36% vs. 22%; *P* < 0.0001), and had a higher Charlson co‐morbidity index score (3.4 ± 1.5 vs. 3.2 ± 1.6 points; *P* < 0.047) compared with patients with a normal FFMI.

Handgrip weakness occurred most often in patients with disease stage II (29% stage I, 40% stage II, 23% stage III; *P* = 0.003). A low FFMI occurred most often in patients with disease stages I and II (32% stage I, 35% stage II, 25% stage III; *P* = 0.038).

Combined handgrip weakness and low FFMI was found in 92 patients (10% of total sample). Handgrip weakness was identified in 151 patients (16%) with normal FFMI, while low FFMI was seen in 162 patients (17%) without handgrip weakness. The majority of patients (*n* = 531; 57%) presented neither handgrip weakness nor low FFMI.

### Overall survival

Median follow‐up was 60 months (range 26–60), and at the time of analysis, 683 patients (73%) had died. Median OS (95% CI) for the whole cohort was 23 months (95% CI: 21–25).

#### Patients with World Health Organization Performance Status 0–1

At baseline, the majority of patients (*n* = 794; 85%) exhibited WHO PS 0 or 1. Median OS was 24 months (95% CI: 22–27). In this subsample, handgrip weakness was present in 176 patients (22%), while low FFMI was found in 197 patients (25%).

**Figure 1:**
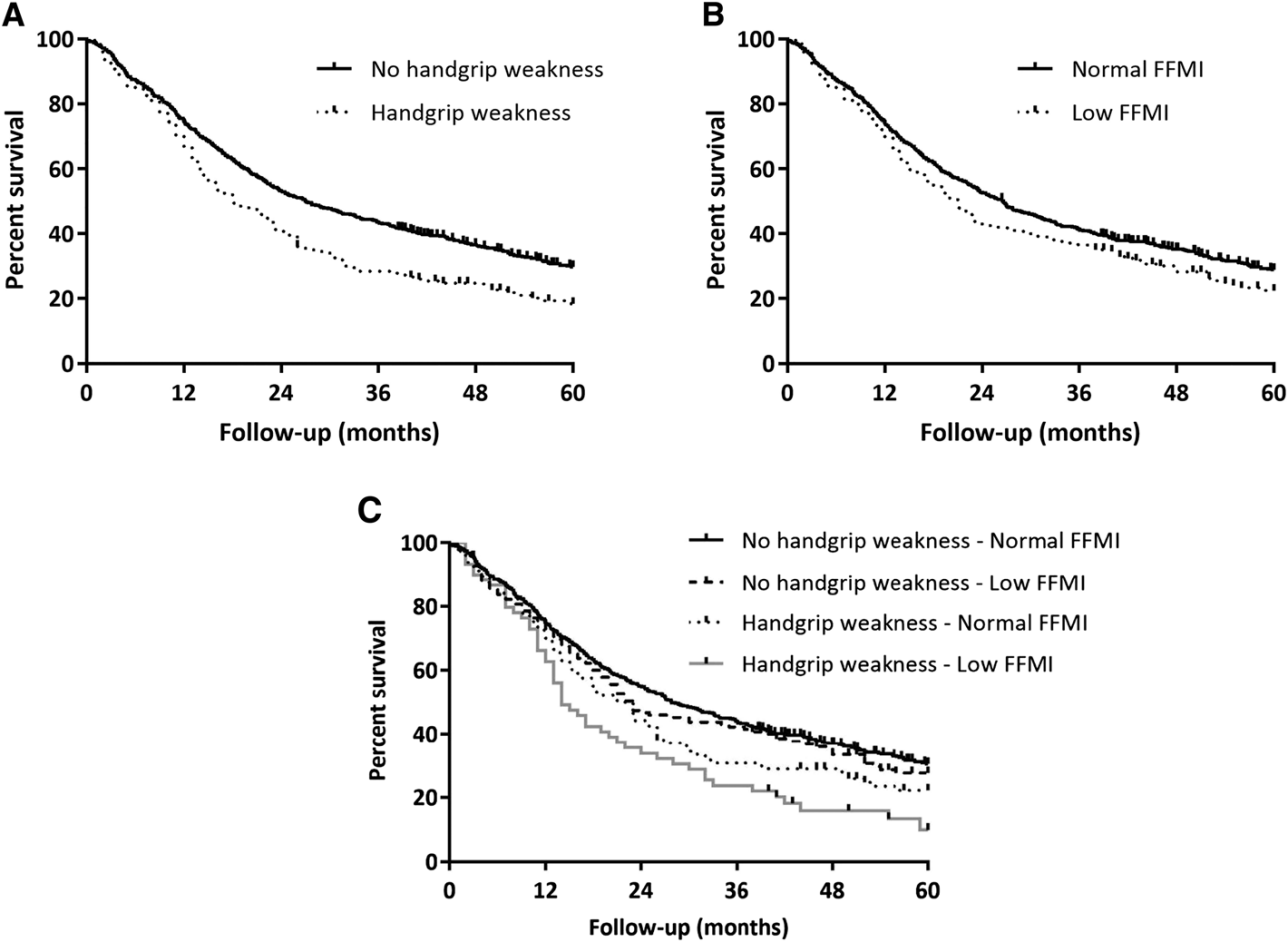
A. Kaplan–Meier survival plots in non‐small cell lung cancer patients with World Health Organization performance score 0 or 1 (*n* = 794): handgrip weakness. B. Kaplan–Meier survival plots in non‐small cell lung cancer patients with World Health Organization performance score 0 or 1 (*n* = 794): low fat‐free mass index (FFMI). C. Kaplan–Meier survival plots in non‐small cell lung cancer patients with World Health Organization performance score 0 or 1 (*n* = 794): combination of handgrip weakness and low fat‐free mass index (FFMI).

Median OS was significantly lower in patients with handgrip weakness compared with patients with normal handgrip strength (OS = 19 months, 95% CI: 15–23 vs. OS = 27 months, 95% CI: 24–32, *P* < 0.0001; HR = 1.43, 95% CI: 1.18–1.74, *P* < 0.001; *Figure* 1A).

Median OS was significantly lower in patients with low FFMI compared with patients with normal FFMI (OS = 21 months, 95% CI: 17–24 vs. OS = 27 months, 95% CI: 23–30, *P* = 0.049; HR = 1.21, 95% CI: 1.00–1.46, *P* = 0.049; *Figure* 1B).

On multivariate analysis, handgrip weakness (HR = 1.31, 95% CI: 1.07–1.59, *P* = 0.008) and low FFMI (HR = 1.24, 95% CI: 1.03–1.51, *P* = 0.024) were associated with OS, when corrected for age, gender, disease stage, and Charlson co‐morbidity index (*Table* 2).

**Table 2:** Univariate and multivariate Cox proportional hazard models

	WHO PS 0 or 1 (*n* = 794)			WHO PS ≥ 2 (*n* = 142)		
	HR	95% CI	*P*‐value	HR	95% CI	*P*‐value
Univariate analysis						
Handgrip weakness^a^	1.43	1.18–1.74	<0.001	1.10	0.77–1.58	0.60
Low FFMI^b^	1.21	1.00–1.46	0.049	1.09	0.76–1.57	0.65
Multivariate analysis						
Handgrip weakness^a^	1.31	1.07–1.59	0.008	1.02	0.69–1.51	0.91
Low FFMI^b^	1.24	1.03–1.51	0.024	1.15	0.78–1.67	0.49
Male gender^c^	1.15	0.95–1.38	0.14	1.23	0.82–1.83	0.32
Age (per year older)	1.01	0.99–1.03	0.06	1.01	0.98–1.04	0.44
Disease stage II^d^	1.36	0.94–1.96	0.11	0.96	0.51–1.80	0.89
Disease stage III^d^	1.82	1.42–2.34	<0.001	2.05	1.32–3.16	0.001
Charlson co‐morbidity index (per point increase)	1.05	0.96–1.14	0.27	1.08	0.92–1.26	0.34
Multivariate analysis combining handgrip weakness and low FFMI						
Handgrip weakness + normal FFMI^e^	1.20	0.94–1.53	0.14	1.25	0.76–2.04	0.38
Normal handgrip strength + low FFMI^e^	1.15	0.91–1.45	0.23	1.43	0.86–2.39	0.17
Handgrip weakness + low FFMI^e^	1.79	1.34–2.40	<0.0001	1.12	0.67–1.86	0.67
Male gender^e^	1.14	0.94–1.37	0.18	1.25	0.83–1.86	0.28
Age (per year older)	1.01	1.00–1.03	0.06	1.01	0.98–1.04	0.40
Disease stage II^d^	1.38	0.95–2.00	0.09	1.00	0.53–1.88	0.99
Disease stage III^e^	1.83	1.42–2.35	<0.0001	2.09	1.35–3.22	<0.001
Charlson co‐morbidity index (per point increase)	1.15	0.91–1.46	0.23	1.07	0.92–1.26	0.38

WHO PS, World Health Organization Performance Status; CI, confidence interval; FFMI, fat‐free mass index; HR, hazard ratio.

a Ref: No handgrip weakness.

b FFMI < 17 kg/m^2^ in male patients and <15 kg/m^2^ in female patients; Ref: FFMI equal to or above these cut‐offs.

c Ref: Female gender.

d Ref: Disease stage I.

e Ref: Normal handgrip strength and normal FFMI.

Sixty‐one patients (8%) presented with a combination of both handgrip weakness and low FFMI. Median OS was significantly lower in patients who showed a combination of handgrip weakness and low FFMI compared with patients with normal handgrip strength and normal FFMI (OS = 15 months, 95% CI: 12–20 vs. 28 months, 95% CI: 24–34, *P* < 0.001; HR = 1.80, 95% CI: 1.34–2.41, *P* < 0.001; *Figure* 1C). Multivariate analysis, including age, gender, disease stage, and Charlson co‐morbidity index, identified the combined presence of handgrip weakness and low FFMI (HR = 1.79, 95% CI: 1.34–2.40, *P* < 0.001) as independent prognostic factor for OS (*Table* 2).

#### Patients with World Health Organization Performance Status > 2

At baseline, WHO PS ≥ 2 was observed in 142 patients (15% of the whole sample). Median OS was 13 months (95% CI: 9–18). In this subsample, handgrip weakness was present in 67 patients (47%), while low FFMI was found in 57 patients (40%).

There was no significant difference in OS between patients with and without handgrip weakness and patients with and without low FFMI (*Table* 2).

## Discussion

To our knowledge, this is the first prospective study addressing the prognostic value of low handgrip strength and low FFMI in patients with NSCLC treated with (chemo)radiation with curative intent. Despite a good WHO PS, 26% and 27% of the patients were identified with handgrip weakness and low FFMI, respectively. In the subset of patients with well‐preserved performance status (WHO PS 0 or 1), both handgrip weakness and low FFMI were independent prognostic factors of OS during 5 year follow‐up. Additionally, patients with combined handgrip weakness and low FFMI exhibited the worst survival rates compared with patients with normal handgrip strength and normal FFMI. Therefore, handgrip weakness and low FFM provide additional objective discriminatory information of patients' prognosis, which complements WHO PS.

It can be speculated that similar results would be obtained in other cancer types in which muscle wasting and weakness are prevalent and have prognostic value, such as colorectal cancer, gastroesophageal cancer, breast cancer, and head‐and‐neck cancer.^18^, ^19^, ^20^ However, the extent to which these findings can directly be translated to other cancer populations remains to be elucidated.

Current strategies mostly employ WHO PS to assess which patients are deemed fit enough to undergo curative treatment. Given the subjective nature of the physician reported WHO PS, it is imperative that additional strategies are employed in order to provide accurate and objective scoring of health status in patients considered for curative treatment. The measurement of handgrip strength and FFM could be an easy and non‐invasive method to provide this additional information. These measurements are quick (e.g. less than 5 min), cheap, and require only minimal training of the assessor. Furthermore, measurement results are immediately accessible for interpretation and record keeping. Reliability coefficients for handgrip strength and bioelectrical impedance are acceptable in sarcopenic populations.^21^, ^22^

Furthermore, our data clearly show that these measurements are able to further detect patient with worse prognosis within a cohort of patients with well‐preserved performance status based on WHO PS score, suggesting that the additional assessment of handgrip weakness and low FFMI during a screening procedure increases its sensitivity to detect vulnerable patients. Whether improved prognostic screening translates into a better tailored individual treatment schedule or effective supportive treatment remains to be elucidated.^23^

One small study examined the value of BIA‐derived FFM and physical performance utilizing short physical performance battery in predicting chemotherapy course completion.^24^ The authors concluded that these tools were helpful for prediction of treatment completion. However, data about survival and WHO PS were lacking.^24^

Few studies have investigated handgrip strength and muscle mass separately in relation to survival. Handgrip weakness has been shown to correlate with survival in several chronic disease conditions [9, 10]. A study in patients with locally advanced, metastatic, or recurrent NSCLC or gastrointestinal malignancy has shown that handgrip strength is also a prognostic factor in the setting of advanced malignancy [6]. However, these authors did not correct their analysis for WHO PS score, which is considered an important prognostic factor, related to handgrip strength.^9^ Handgrip weakness was present in 25% of patients, which is a high proportion compared with a cohort of patients with moderate to very severe chronic obstructive pulmonary disease, in which 15% showed handgrip weakness using the same criteria.^25^

This study found that also nearly one out of every four patients exhibited low FFM prior to start of treatment, which was an independent prognostic marker in NSCLC patients. This is in line with previous literature regarding computed tomography‐derived muscle mass.^26^

The strength of this study comes from its prospective nature and the large sample size. A potential drawback of the study includes the fact that it is a single‐institutional study. Second, FFMI was measured by BIA, which is inferior to FFMI derived from dual‐energy X‐ray absorptiometry or computed tomography,^27^, ^28^, ^29^, ^30^ as it tends to underestimate FFM in patients with cancer.^31^ However, BIA is less expensive and easy to implement. The implementation of fast, cost‐efficient, and feasible measures to quantify body composition is an important challenge in the oncology domain.^32^ Third, co‐morbidities were collected directly from original medical records, but underreporting cannot be excluded. Because co‐morbidities potentially have an impact on muscle tissue,^33^ it is important to inventorize them in a standard manner. Nevertheless, we assume that co‐morbidities that have a major impact on patients' condition and treatment have been noted. Fourth, this study only included patients that were selected for curative‐intent (chemo)radiation. Therefore, selected patients with disease stages I and II were more likely to have a lower performance status compared with a random sample of patients with disease stages I and II. This is reflected by the higher frequency of handgrip weakness and low FFMI in these patients groups compared with patients with disease stage III. Fifth, we did not record patterns of recent weight loss in our patients, which is an established and easy to assess prognostic factor in patients with cancer.^34^

Recently, a revised European consensus on definition and diagnosis of sarcopenia has been published.^35^ This guideline proposes a single cut‐off for each gender to define handgrip weakness as a screening tool for sarcopenia next to body composition. Given the observation that height, age, and gender are strongly related to handgrip strength in a sample 502 713 healthy adults,^12^ we opted to use individually defined cut‐off values to define handgrip weakness.

In conclusion, these results indicate that handgrip weakness and low FFMI are independent prognostic factors for OS in patients with early and locally advanced NSCLC treated with curative‐intent (chemo)radiation. They might be of particular interest in the large subset of patients with well‐preserved performance status according to the treating physician. Patients with combined handgrip weakness and low FFMI clearly have a worse prognosis in this patient group.

## Conflict of interest

All authors declare that they have no relevant conflict of interest.
